# Identification of Potential Lead Molecules for Zika Envelope Protein from In Silico Perspective

**Published:** 2019

**Authors:** Selvaa Kumar Chellasamy, Shine Devarajan

**Affiliations:** Faculty of Biotechnology and Bioinformatics, D.Y. Patil Deemed to be University, CBD Belapur, Navi Mumbai, India

**Keywords:** Aedes, Dengue virus, Envelope protein, Flavivirus, Microcephaly, Zika virus

## Abstract

**Background:**

Zika virus is the family member of flavivirus with no reported clinically approved drugs or vaccines in the market till date. This virus is spread by *Aedes* mosquitoes, and can also be transmitted through sexual contact or blood transfusions. There are reported medical conditions like microcephaly among new-borns delivered by infected pregnant women. The envelope protein of Zika virus is associated with virulence, tropism, mediation of receptor binding and membrane fusion. ED1-EDII domain (K1 loop pocket) is an integral part of the envelope protein and a potential drug target. In the present study, the purpose was to identify the potential lead molecules to dock against K1 loop which could be later considered as flavivirus entry inhibitors.

**Methods:**

Multiple sequence alignment method was considered for the analysis of indels in envelope protein. Phylogenetic tree was constructed based on the alignment. Aliphatic index, GRAVY scores and hydropathy plot of the envelope proteins were calculated for the flavivirus family members. Zika envelope protein was homology modeled and considered for protein-ligand docking analysis with chemical compounds of known functions.

**Results:**

As per in silico based analysis, the envelope protein of Zika virus is highly hydrophilic with the least number of amino acid deletions compared to rest of the family members. During docking studies, it was observed that compounds like NITD, compound 6, P02, Doxytetracycline and Rolitetracycline show better binding affinity with Zika envelope protein compared to dengue virus.

**Conclusion:**

These better binding compounds could be the promising lead molecules for Zika envelope protein which could better block the viral entry.

## Introduction

Zika virus (ZIKV) belongs to *flavivirus* family which was first isolated from a sentinel rhesus monkey in the Zika Forest, Uganda in 1947 1. Human population gets infected by the bite of an infected *Aedes* species 2. In recent years, Brazil has reported Zika viral infection at a larger scale 3. Even Iran is highly exposed to the Zika infection due to favorable environment and the presence of three members of *Aedes* genus 4,5. Common symptoms associated with the infection are fever, skin rashes, conjunctivitis, joint pain, malaise and headache which are quite similar to that of dengue and chikun-gunya 6,7. Other members of this family include West Nile, dengue, yellow fever, and Japanese encephalitis 8,9. A recent study states that this viral infection can be sexually transmitted and can also be passed on from the pregnant women causing micro cephaly among the new borns 10,11. Interestingly, *Aedes* mosquitoes remain the common vector for transmitting both dengue and Zika virus 2. Till date, no drugs or vaccines were reported against this viral infection 7. The serious threat of viral infection has recently hard-pressed WHO to declare a global public health emergency 12.

The completely sequenced Zika viral genome is available online with 10,272 nucleotides 12–14. This gets translated into a single polyprotein. Furthermore, they were post and co-translationally cleaved by both host and viral proteases much like rest of their family members 15. This polyprotein comprises three structural (capsid, envelope and premembrane) and seven non-structural proteins (NS1, NS2A, NS2B, NS3, NS4A, NS4B, NS5) 16–18. These structural proteins were reported to be involved in the formation of the viral particle 19. The non-structural proteins were involved in flavivirus assembly 20. The present study focused on the envelope protein (E protein) which is considered as the major determinant of virulence, tropism since it plays a critical role in mediation of receptor binding and membrane fusion 21–24.

The N-terminal region of E protein contains three well characterized domains as determined by crystallographic studies which include ED1, EDII and E-DIII 25–28. As such, EDI has no reported functions in ZIKV which is otherwise required for viral entry into the host cell in other flaviviruses. Furthermore, EDII contains a hydrophobic fusion loop at its distal end which is a dimerization domain. It is proposed to bind to the membrane of the endosome to facilitate fusion between virus and endosomal membrane. EDIII with immuneglobulin fold participates in both receptor binding and fusion. In particular, EDI-EDII combo forms a hydrophobic fusion loop (K1 pocket loop) which happens to be a potential drug target. As per the crystal structure report, the hinge angle between ED1 and EDII varies among the family members. This was found to be highly flexible and is required for flexing of the EI-DII during the fusion process in order to expose the fusion loop 24,29,30.

In dengue envelope protein (DENV), EI-EDII combo interacts with β-N-octylglucoside (β-OG) which in turn can sterically hinder the conformational change between these domains which is essential for virus-host membrane fusion 24. A single glycosylation site (Asn 154) was observed in Zika envelope protein (ZIKV E) which is two (Asn67 and Asn153) in DENV E. It has also been reported that the amino acids surrounding Asn154 differ in ZIKV E and in other flavivirus, which may provide insight into the pathobiology of Zika virus 31. The C-terminal region of E protein consists of two alpha helices (EH1 and EH2) in the stem region and two helices in the transmembrane region (ET1 and ET2). Both ET1 and ET2 are associated with the assembly of E-protein 32–34. The main objective of this study was to identify the potential lead molecules for the ZIKV E protein which shows better interactions with ED1-EDII domain from in silico perspective. This study explored the molecular level interactions of ZIKV E protein with the leads which is not feasible with the conventional method. These compounds could be further used against ZIKV E protein for therapeutics and thus can arrest the virus recognition to the host cell.

## Materials and Methods

### Sequence analysis

The complete genome polyprotein of Zika virus, West Nile, yellow fever, Japanese encephalitis and dengue was downloaded from Uniprot Database 35, [Q 32ZE1: Zika; P27395: Japanese encephalitis; P06935: West Nile; P17763: Dengue; P03314: Yellow fever] 35. Out of these, only the envelope protein domains were extracted from all five organisms. These envelope proteins were subjected to sequence analysis using Protparam software 36 wherein, the purpose was to study their overall aliphatic index and GRAVY score (Grand Average of hydropathy). Basically, Aliphatic Index method 37,38 predicts regional stability by calculating the relative volume occupied by aliphatic side chains. This is a positive indicator of globular protein thermostability. However, the GRAVY value is calculated by adding the hydropathy value for each residue and dividing them by the length of the sequence by using Kyte-Doolittle method 39. This can be calculated as the sum of the hydropathy values for all the amino acids in a protein divided by the total number of residues in it. Next, all five sequences were considered for hydrophobicity analysis using Kyte-Doolittle hydropathy plot 39. It is a quantitative analysis of the degree of hydrophobicity or hydrophilicity of amino acids of a protein which is used to characterize or identify possible structure or domains of a protein. The graph above zero defines them as hydrophobic whereas below zero is considered as hydrophilic. Finally, all five sequences were considered for multiple sequence alignment using Clustal Omega software 40. A phylogenetic rooted tree (Neighbour Joining method) was constructed based on the multiple alignment to identify the close homolog of Zika virus.

### Homology modeling

Search for crystal structure of ZIKV E protein has listed nine structures docked with antibody in Protein Data Bank (PDB) (https://www.rcsb.org/pdb/home/home.do) (PDB ids: 5JHM, 5JHL, 5KVD, 5KVE, 5KVG, 5KVF, 5VIG, 5GZN and 5GZO) 41–44. All these crystal structures were available in post-fusion form with closed ED1-EDII loop. These structures cannot be considered for docking due to their closed hydrophobic pocket. Further search within PDB has listed a pre-fused crystal structure of dengue with open hydrophobic pocket (PDB id: 1OKE). This conformation was due to the local rearrangement of the K1 beta hairpin between residues 268–280. Here, the envelope protein is in complex with n-octyl-beta-D-glucoside within the hydrophobic pocket 24. To generate the similar conformation in ZIKV E, homology modeling was opted using SWISSMODEL server 45. Finally, generated model was energy minimized using Swisspdbviewer software 45 and validated using Ramachandran plot using RAMPAGE 46 and PROSA (Protein Structure Analysis) software 47.

### Protein-ligand docking

Based on literature review, 10 ligands [A1-A5, NIT-D, compound-6 (with a quinazoline core), P02, Doxytetracycline (with tetracyclic ring structure) and Rolitetracycline (with tetracyclic ring structure)] were downloaded from Maybridge chemical database 48. These compounds have a significant biological affinity (*μM*) with DENV shown in table 1. In particular, compound A4 and A5 showed good antiviral activity in DENV 49–53. Their physiochemical properties are listed in table 2. Mostly, they are thiazole derivatives critically involved in arresting viral replication in cell-based assays 54. These chemical compounds are available in 2D form, which were converted into 3D conformers using ChemAxon (http://www.chemaxon.com) software. The modeled ZIKV E protein was considered as receptor for docking against these ten ligands using AutoDock software (Version 4.2) 55. In the parameters section, Lamarckian genetic algorithm was selected as a scoring function for identifying the favorable conformation in the binding site. A grid box was constructed at the interface of D1-DII domain of the receptor with a map dimension of 30×30×30 and kept 1 *Å* grid spacing for accommodating all the amino acids in the binding site. The grid center of the x y z box coordinates were set as −8.141, 80.423 and 45.672, respectively. Based on the above settings, AutoGrid parameters for each ligand within the binding sites were calculated. After successful generation of each grid box, Lamarckian genetic algorithm based docking parameters were prepared to generate the conformations of the ligands. A population size of 150 was used for generating 50 conformations for each ligand with a maximum number of 2500000 evaluations per cycle. The rate of gene mutation and crossover parameters in the algorithm were set as 0.02 and 0.8, respectively. Among 50 conformations, the most favorable compound was selected based on their binding affinity. Similar steps were followed for docking dengue envelope protein with the ten ligands as a reference.

**Table 1.** The structure and activity of 10 compounds (*μM*) against DENV envelope protein

**No.**	**Compound**	**Structure**	**Activity against DENV (*μM*)**	**Reference**
**1.**	A1	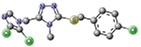	>100 ^*^	(46)
**2.**	A2	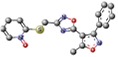	>100 ^*^	(46)
**3.**	A3	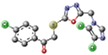	>100 ^*^	(46)
**4.**	A4	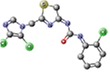	32±17 ^*^	(46)
**5.**	A5	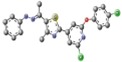	1.2±0.7 ^*^	(46)
**6.**	Compound-6	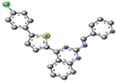	0.119/>20 ^#^	(47)
**7.**	NITD-448	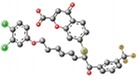	9.8/48.7 ^#^	(48)
**8.**	P02	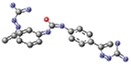	13/371 ^#^	(49)
**9.**	Doxycycline	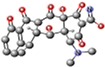	55.6/>500 ^#^	(50)
**10.**	Rolitetracycline	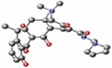	67.1/>500 ^#^	(50)

* IC_50_ (*μM*),

# (EC_50_/CC_50_)

**Table 2.** Physicochemical properties of ten chemical compounds

**Compound**	**Hydrophobicity LogP**	**Aquous solubility LogS**	**Blood brain barrier (BBB)**	**Human Intestinal absorpotion (HIA)**	**PgP substrate/Inhibit or**	**Acute oral toxicity class**	**Caco2 permea bility (LogPapp,*cm/s*)**	**Rat acute toxicity (LD50, *mol/kg*)**
**A1**	3.67	−3.88	+	+	No	III	1.50	2.37
**A2**	3.70	−4.01	+	+	No	III	1.42	2.38
**A3**	4.12	−3.83	+	+	No	III	1.64	2.50
**A4**	4.39	−4.24	+	+	No	III	0.97	2.50
**A5**	6.77	−6.12	+	+	No	III	1.32	2.19
**Compound-6**	5.86	−6.22	+	+	No	III	1.31	2.43
**P02**	3.57	−4.44	+	+	No	III	0.98	2.50
**NITD**	6.44	−6.58	+	+	No	III	0.50	3.07
**Doxytetracycline**	1.14	−2.37	+	+	No	III	1.40	2.51
**Rolitetracycline**	1.48	−2.55	−	+	No	III	0.68	2.78

## Results

### Sequence analysis

**Figure 1.**
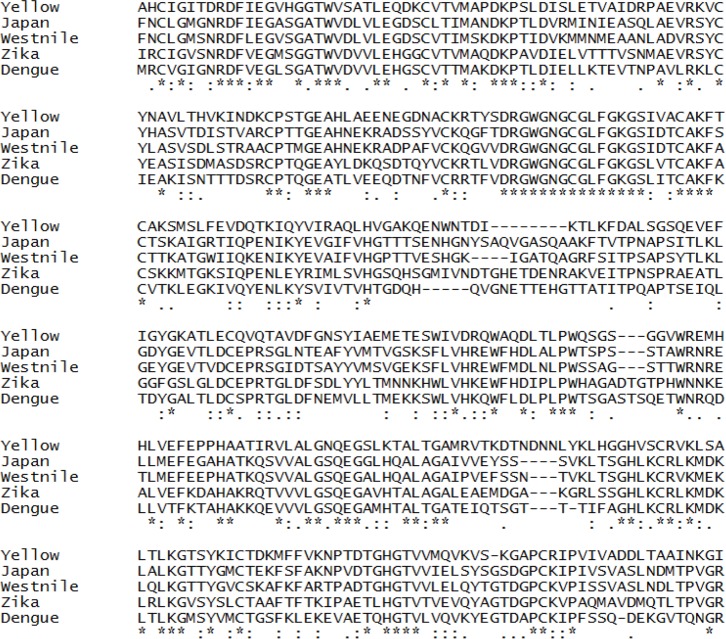
Multiple sequence alignment of yellow fever, Japanese encephalitis, West Nile, Zika and dengue envelope proteins. The identical, conservative and non-conservative substitutions are shown as asterisk (*), colon (:) and dot (.), respectively. Deletions were denoted as hyphen (-).

**Figure 2.**
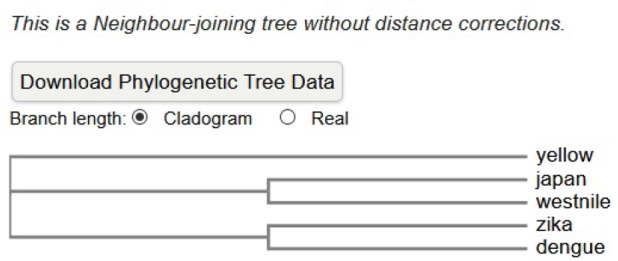
The pairwise alignment of the K1 loop of dengue and Zika envelope proteins (268–280) highlighting the region of identical and semi-conservative residues which are critically involved in drug interaction.

**Figure 3.**
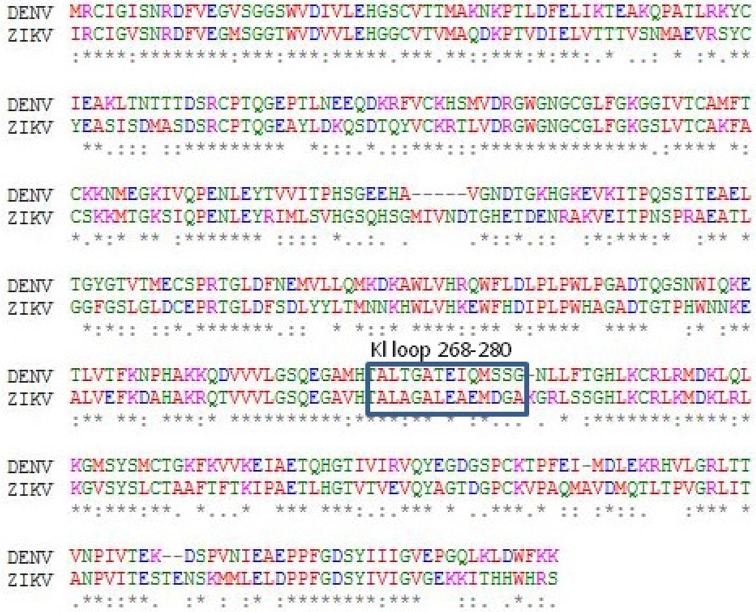
The rooted phylogenetic tree generated using Clustal Omega software for Japanese encephalitis (P27395), dengue (P17763), West Nile (P06935), yellow fever (P03314) and Zika (Q32ZE1) envelope proteins.

**Figure 4.**
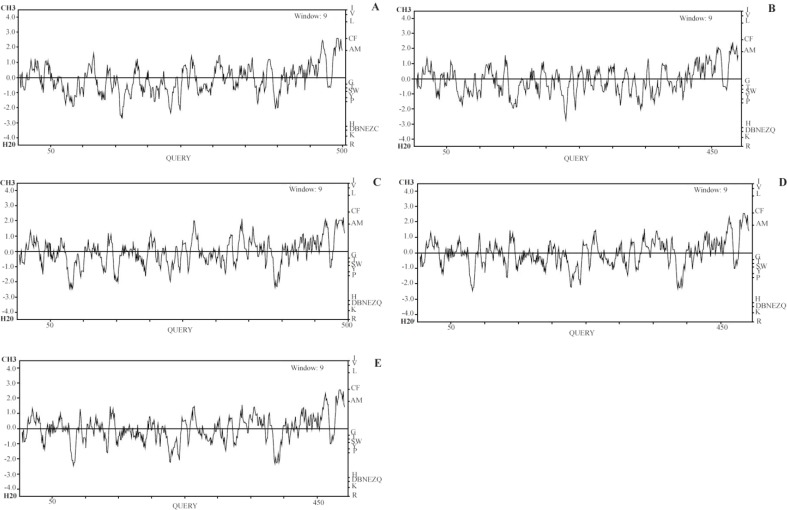
Hydrophobicity analysis using Kyte-Doolittle hydropathy plot for envelope proteins of (a) Zika (b) dengue (c) Japanese encephalitis (d), West Nile (e) and yellow fever.

Multiple sequence alignment of all five envelope proteins reveals that minimum number of deletions were observed in Zika and maximum in yellow fever (Figure 1). Based on this alignment, a phylogenetic rooted tree (Neighbour Joining) was generated wherein Zika and dengue envelope proteins share a common internal node. Thus, these two OTUs (Operational Taxonomic Unit) are close homologs. Other OTUs, like Japanese encephalitis and West Nile share a common ancestral internal node. However, yellow fever is the outlier (Figure 2). A separate alignment of the K1 loop (268–280 of 13 residues length) of Zika and dengue confirms six identical residues followed by two conservative and three non-conservative substitutions (Figure 3). Next, the GRAVY score (Grand Average of hydropathy) was calculated for all five E proteins. The ZIKV E protein is highly negative followed by dengue virus. However, West Nile has a positive GRAVY score. Conversely, aliphatic index value was calculated. Yellow fever has the maximum index value and Japanese encephalitis has the minimum (Table 3). Finally, Kyte-Doolittle hydropathy plots were generated for all five envelope proteins. In these plots, Zika and dengue virus falls below the zero which confirms them as hydrophilic in nature. However, most of the segments of West Nile, Japanese encephalitis and yellow fever moves above zero marking them as hydrophobic (Figures 4A–E).

**Table 3.** Comparison of GRAVY and Aliphatic Index score of envelope proteins of Zika, dengue, Japanese encephalitis, West Nile and Yellow fever

**Virus**	**Swissprot accession number**	**Envelope protein**	**GRAVY score**	**Aliphatic index score**
**Zika**	Q32ZE1	291–790	−0.078	82.22
**Dengue**	P17763	281–775	−0.052	85.23
**Japanese encephalitis**	P27395	295–794	−0.008	80.56
**West Nile**	P06935	291–787	0.025	82.19
**Yellow Fever**	P03314	286–778	−0.020	85.98

### Homology modeling

**Figure 5.**
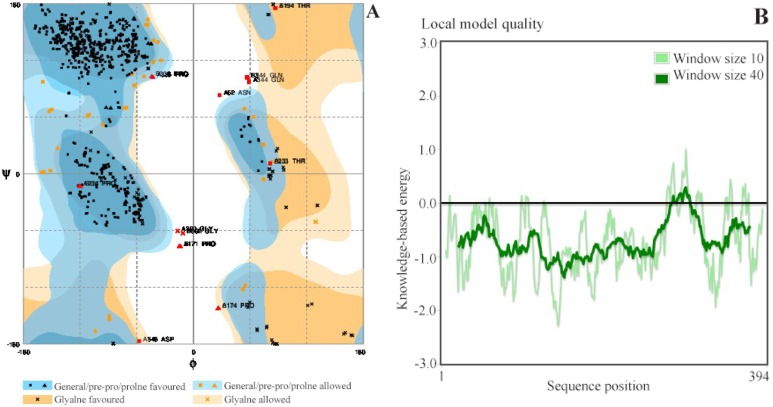
A) Ramachandran plot of modeled Zika envelope protein. B) PROSA showing the local model quality of the modeled Zika protein. The plot is generated with a window size of 40.

Availability of post-fused crystal structures of ZIKV E protein has compelled homology modeling to generate an opening conformation of the K1 loop. Thus, the 3D structure of DENV E protein (PDB id: 1OKE) with a bound n-octyl-beta-D-glucose was considered as the template for modeling ZIKV E protein. From sequence perspective, both proteins share 55.87% identity making them a perfect template for modeling. Modeled structure was energy minimized and considered for model validation. As per the Ramachandran plot analysis, only six residues were observed in the disallowed region (Figure 5A). Mostly, residues were within the allowed region. As per PROSA report, most of the regions were lying below zero confirming their overall structural stability (Figure 5B).

### Protein-ligand docking

**Figure 6.**
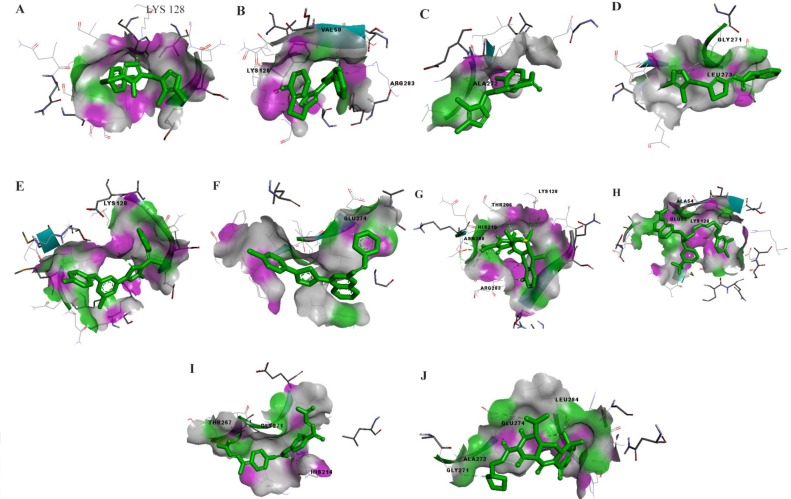
The docked poses of ligands A1–A5 with the envelope protein of dengue (a–e). The interactions between compound 6, Doxycycline, NITD, PO2 and Rolitetracycline (f–j). The purple color denotes hydrogen bond donor and the green color denotes hydrogen bond acceptor.

**Figure 7.**
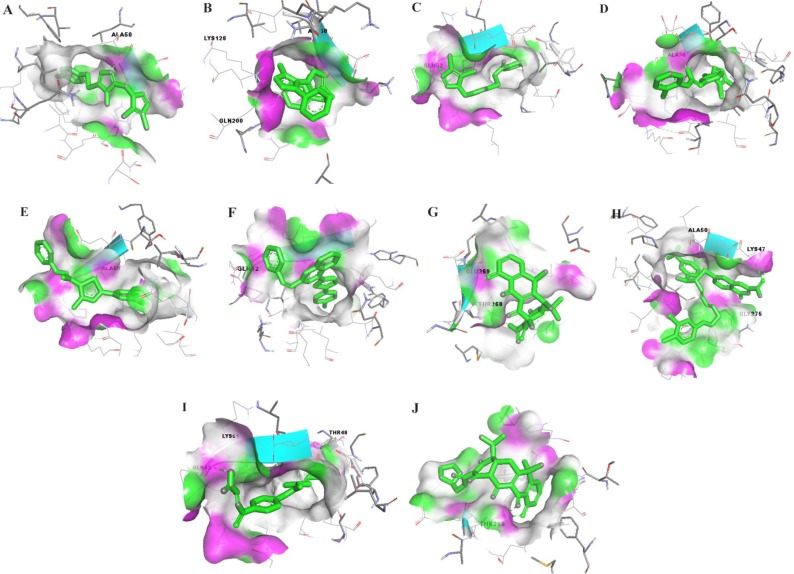
The docked poses of ligands A1–A5 with the envelope protein of Zika (a–e). The interactions between compound 6, Doxycycline, NITD, PO2 and Rolitetracycline (f–j). The purple color denotes hydrogen bond donor and the green color denotes hydrogen bond acceptor

Modeled ZIKV E protein was docked with ten chemical compounds. The K1 loop of Zika envelope protein exhibits better protein-ligand interaction with compound-6, Doxycycline, NITD, P02 and Rolitetracycline. However, compounds like A1-A5 show lesser binding affinity (Figures 6A–J). In contrast, the K1 loop of DENV E protein shows better interaction with A1-A5 compounds as reported earlier in the literature 49. Furthermore, compound-6, Doxycycline, NITD, P02 and Rolitetracycline display lesser binding affinity (Figures 7A–J) (Table 4). The RMSD scores for A1 to A5 between DENV and ZIKV are 2.386Å, 1.532Å, 2.961Å, 1.838Å and 2.338Å, respectively. However, compound-6, Doxycycline, NITD, P02 and Rolitetracycline show large deviation in their values due to the distinct binding behavior between the two viral proteins.

**Table 4.** The docking score of ten compounds docked against dengue and zika envelope protein

**S.No**	**Chemical Compounds**	**Dengue**		**Zika**	

		**Amino acids**	**Binding energy (*kcal/mol*)**	**Amino acids**	**Binding energy (*kcal/mol*)**
**1**	A1	Ala50	−4.25	Lys128	−1.27
**2**	A2	Ala50, Lys128, Gln200	−2.8	Lys128, Val50, Arg283	−2.23
**3**	A3	Gln52	−3.32	Ala272	−1.16
**4**	A4	Ala50	−3.68	Gly271, Leu273	−2.54
**5**	A5	Ala50	−4.48	Lys128	−1.14
**6**	Compound6	Gln62	−1.4	Glu274	−2.17
**7**	Doxycycline	Gln269, Thr268	−4.14	Thr205, Lys128, His210, Asn208, Arg283	−5.93
**8**	NITD	Ala50, Lys47, Gly275	−3.92	Ala54, Glu55, Lys128	−6.25
**9**	P02	Gln62, Lys51, Thr48	−3.32	Thr267, Gly271, His214	−5.20
**10**	Rolitetracycline	Thr268	−3.34	Gly271, Ala272, Glu274, Leu284	−5.21

## Conclusion

Outbreak of Zika viral infection and non-availability of drug therapeutics against this disease have compelled identification of better lead molecules. In this study, envelope protein of ZIKV was the main focus with reference to rest of their family members. ZIKV E protein is quite distinct from rest of them with the least number of amino acid deletions. Furthermore, from phylogenetic perspective, they are closer to DENV E protein. Based on this report, the chemical compounds better binding with DENV E proteins were docked with ZIKV E protein. Here, it was observed that compounds like NITD, compound-6, P02, Doxytetracycline and Rolitetracycline showed better binding affinity with ZIKV. Irrespective of being structurally similar, lead interactions differ between DENV and ZIKV proteins which was evident through their binding affinity and RMSD score. Thus, it can be concluded that these five small molecules which were exhibiting better interaction with ZIKV E protein could be promising lead molecules. A wet lab based study could assist in understanding the role of these molecules in blocking the function of viral envelope proteins to prevent viral entry.
